# Is the surface topography a helpful tool for the management of scoliosis?

**DOI:** 10.1186/1748-7161-7-S1-O17

**Published:** 2012-01-27

**Authors:** D Papadopoulos

**Affiliations:** 1 SPONDYLOS Laser Spine Lab , Athens, Greece

## Background

The aim is to reveal the importance of surface topography in complement to the x-rays [1,2].

## Material and methods

We have used the Formetric 4D-Dicom II system, which is supplied also with lateral Cobb angle measurement. We have examined 616 patients (432 females and 184 males), age 5 y to 21 y. The patients have been visited clinically by inspection, Adams forward bending test and Perdriolle scoliometer. We have fit, to every patient, 4-8 reflectors on the apex of T1 through L4 spinal process and 2 shoulder reflectors to get the possibility for lateral Cobb angle measurement with the Formetric 4D.

## Results

We had various data through surface topography. Torsion, rotation, shoulder tilt, etc, but we have insisted on the Cobb angle measurement. We had a >95% accuracy in Scoliosis between 22° and 65° Cobb angle. The accuracy was lower, between 95% to 70%, if the measured curve was > 65° and very poor, less than 50%, when the curve was < than 20° in x-rays. As it concerns the Kyphosis, the cob angle was very accurate as it was exceeded 90%.

## Conclusions

We believe that the surface topography is a precious tool for the diagnosis and follow up of a complex three dimensional skeletal deformity, such as scoliosis. The accuracy of the Cobb angle measurement is excellent and we believe that we must move to the next step, which is the 3D dimension.
